# Effects of High Intensity Ultrasound Stimulation on the Germination Performance of Caper Seeds

**DOI:** 10.3390/plants12122379

**Published:** 2023-06-19

**Authors:** María Laura Foschi, Mariano Juan, Bernardo Pascual, Nuria Pascual-Seva

**Affiliations:** 1 Departament Producció Vegetal, Universitat Politècnica de València, 46022 Valencia, Spain; mafos@doctor.upv.es (M.L.F.); mjuan@upv.es (M.J.); bpascual@upv.es (B.P.); 2 Horticulture and Floriculture, Agriculture Faculty, National University of Cuyo, Mendoza M5528AHB, Argentina

**Keywords:** *Capparis spinosa*, dormancy, holding time, output power, seed coat disruption, ultrasonic probe, viability, water uptake

## Abstract

The caper bush has developed different mechanisms to survive in Mediterranean conditions, such as drought tolerance and seed dormancy. Many studies have been carried out to improve the germination of caper seeds, but ultrasound is one of the least studied methodologies in this species. This study aimed to analyze the effects of treatments with an ultrasonic probe processor on the imbibition and germination of caper seeds. After applying the ultrasound treatment using three output powers and three holding times, the seed coat’s disruption level was determined, and the imbibition, viability and germination tests were carried out. Ultrasonication fastens the initial imbibition, but after 48 h of soaking, seed moisture does not present differences compared to non-sonicated seeds. It produces the scarification of the testa but does not affect the tegmen, so moistening occurs through the hilar region, as in control seeds. There is a significant linear and negative correlation between the germination of the seeds and the temperature reached during the sonication treatment, so that temperatures above 40 °C practically annulled the germination. The combination of 20 W and 60 s provided the greatest germination percentage, being the only treatment that statistically improves germination in relation to the control seeds. When the output power and/or holding time were higher, the temperature increased, and the germination percentage statistically decreased.

## 1. Introduction

The caper (*Capparis spinosa* L.) bush is a perennial shrub naturally widespread throughout the Mediterranean Basin [1]. It has developed different survival mechanisms in Mediterranean conditions [2], such as drought tolerance and seed dormancy. Even though the fruits contain many seeds, they have a very low germination percentage, which is a problem for the cultivation of capers.

The caper seed coat is impermeable and hard to the touch; the testa and tegmen form it, presenting two anatomical structures, the hilum and the micropyle. The testa consists of a layer of 1–2 thick cells with lignified and thickened walls. The tegmen presents a lignified exotegmen composed of several brachysclereid cells and a lignified, fibrous endotegmen composed of a few layers of cells [3,4].

Many studies have tried to improve caper seed germination and to break the possible physical dormancy with different types of scarifications (mechanical, chemical, thermal or biological) [5,6,7,8] and to break the physiological dormancy with the use of gibberellic acid (GA_3_) and potassium nitrate [9,10,11,12]. The ultrasound application has been used in seed germination studies of both cultivated plants and weeds [13,14,15,16,17,18], but it is one of the least-studied methodologies in caper seeds.

The term “ultrasound” describes the sound waves whose frequencies are over the upper audible limit of human hearing in healthy young adults (20 kHz). Sonication is the act of applying sound energy to agitate particles in a sample. When ultrasonic frequencies are used, the process is known as ultrasonication [19].

The effects from the ultrasonication of liquids are caused by cavitation. By introducing high power ultrasound into a liquid medium, the sound waves are transmitted in the fluid and create alternating high-pressure (compression) and low-pressure (rarefaction) cycles, with rates depending on the frequency. High-intensity ultrasonic waves create small vacuum bubbles in the liquid during the low-pressure cycle. When the bubbles attain a volume at which they can no longer absorb energy, they collapse violently during a high-pressure cycle. This phenomenon is termed cavitation [20].

For ultrasonic applications, both ultrasonic probes and ultrasonic baths can be used. The probe sonication is more effective and powerful than the ultrasonic bath in the nanoparticle’s dispersion; the ultrasonic bath device provides a weak ultrasonication and a non-uniform distribution, while the ultrasonic probe device provides a strong ultrasonication and a uniform distribution [21]. The intense sonication zone is directly beneath the probe (sonotrode) when samples are sonicated using an ultrasonic probe device; thus, the ultrasonic irradiation distance is limited to a specific area of the sonotrode’s tip. Ultrasonic processes in open beakers are mainly used for sample preparation of small volumes.

The bubble collapse leads to an increase on the liquid temperature, and the pressure differences may mechanically damage the cellular and tissue structures of the seeds. If the bubbles collapse close to the seed coat, its surface may be damaged, generating pores. If the seed coat represents a physical barrier reducing the water and oxygen uptake into the inner parts of the seed [22], an increase in its porosity could increase the water and oxygen intake, and, in consequence, in germination [14]. However, if the liquid temperature or the pressure differences are excessive, the damage exerted on the tissues of the seed can negatively affect the embryo, decreasing germination. Mechanical actions, such as cutting or removing the seed coat, accelerate the germination process in caper seeds [4], so a similar result is foreseeable due to the hypothetical opening of pores that could occur in the seed coat with the application of the ultrasonication treatment, as occurred in other species. Ultrasound waves application led to greater and faster germination in barley seeds [13], increased germination percentages of deteriorated *Arabidopsis thaliana* seeds [14], accelerated the germination progress, increasing the germination rates of rice seeds [23], and induced dormancy break in *Chenopodium album* [15].

Given these positive results obtained with ultrasonication in other species, this study aimed to analyze the effects of the application of treatments with an ultrasonic probe processor on the imbibition and germination of a caper seed lot. This seed lot was own-produced by the research team, and it was known that its germination percentage was moderate, similar to that of the other own-produced lots used in previous studies.

## 2. Results and Discussion

### 2.1. Ultrasonication

The calibration resulted in the corresponding output powers for 25%, 50% and 75% amplitudes to be 20, 50 and 100 W, respectively. Regarding the temperature, the starting point was the ambient temperature (20 °C) increasing as both the output power and the holding time (exposure time) increased, reaching over 80 °C at maximum amplitude and time (75% (100 W) for 300 s). Similarly, the electrical conductivity (EC) increased as the output power and the holding time increased. At the same time, the water used in the ultrasonication acquired a brown hue, becoming darker as the output power and holding time increased (Figure S1), probably due to partial degradation of the seed coat. 

**Figure 1:**
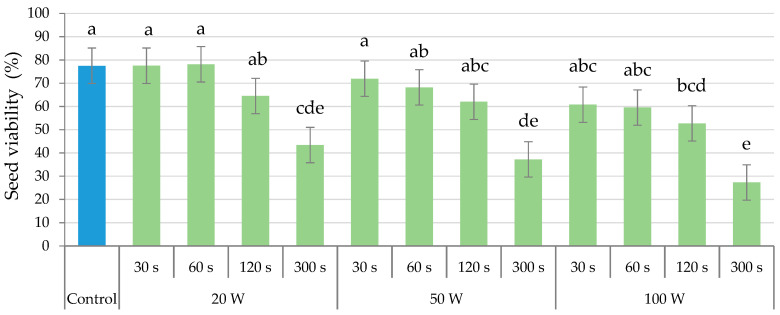
Effect of ultrasound treatments at different output powers (20, 50 and 100 W) and holding times (30, 60, 120 and 300 s) in the seed viability. Different letters indicate significant differences according to the LSD test (*p* ≤ 0.05), which are represented by the error bars.

Figure 1 shows the effect of the ultrasound treatment on the viability of the seeds for the different output powers and holding times applied in the calibration test. The viability of the seeds stimulated for 300 s, with any of the three amplitudes tested, was statistically lower (*p* ≤ 0.05) than those of the control seeds, showing a tendency to decrease with increasing output power (although not statistically significant). Regarding the other holding times, only the application of the 100 W during 120 s statistically reduced the seed viability (*p* ≤ 0.05) compared to the non-treated seeds.

**Figure 2:**
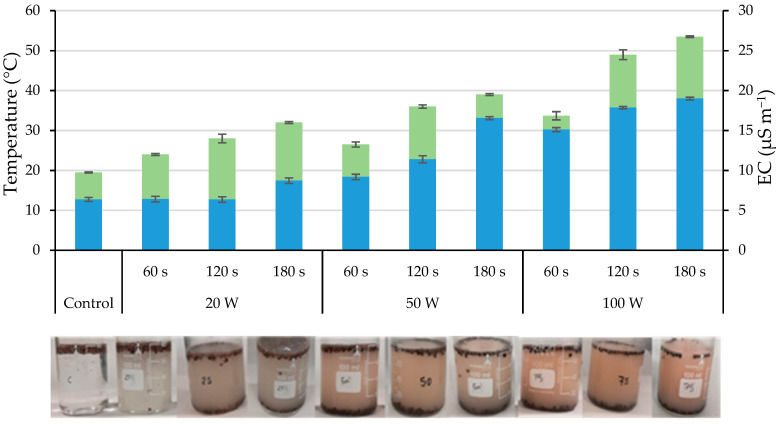
Temperature (green) and electrical conductivity (EC; blue) of the water after ultrasonication of the seeds with different output powers (20, 50 and 100 W) and holding times (60, 120 and 180 s). Error bars represent the standard error (**above**). Appearance of the water used in the corresponding ultrasonication (**bass**).

Given the low viability of seeds stimulated during 300 s, probably related to the high temperature reached and possibly the damage produced in the seed coat, it was decided to discard this holding time, trying to keep the temperature below 60 °C. Thus, to carry out the experiment, the seeds were ultrasonically stimulated using the sonicator with holding times of 60, 120 and 180 s and with the three already assayed output powers (20, 50 and 100 W). These powers are lower than those used in seed hydration and germination studies such as chickpea and rice, in which powers up to 400 W were used [16,24]. Figure 2 presents the temperature and EC registered in the water after applying the ultrasounds at different output powers and holding times

The initial water temperature (20 °C) increased with both the increasing output power and holding time, ranging from 24 °C (20 W, 60 s) to 53.5 °C (100 W, 180 s). The ultrasonication treatments of 20 W both during 60 s and 120 s did not alter the water EC (6 µS m^−1^), but it increased by increasing both the output power and the holding time, up to 19 µS m^−1^ at 100 W for 180 s (Figure 2). At the same time, the water used in the ultrasonication acquired a brown tone, becoming darker as both the output power and the holding time increased (Figure 2). It should be noted that by increasing the holding time and/or the output power of the ultrasonic treatment, a higher percentage of seeds moved to the bottom of the beaker, indicating that the density of these seeds had increased during the treatment because during this short period, further imbibition had occurred in them.

### 2.2. Disruption of the Seed Coat

**Figure 3:**
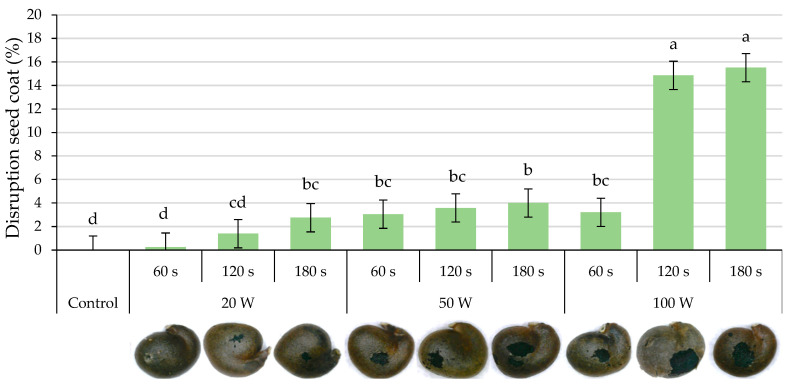
Disruption of the seed coat. Percentage of scarified area in seeds treated with the sonicator at different output powers (20, 50 and 100 W) for 60, 120 and 180 s. Different letters indicate significant differences according to the LSD test (*p* ≤ 0.05), which are represented by the error bars (**above**). Seed coat appearance after ultrasonication (**bass**).

Figure 3 shows the damage produced in the seed coat (expressed as a percentage of the affected surface compared to the seed surface) due to the different treatments (output power and holding time). The scarification of the testa is observed, while the tegmen is visible in the scarified area. The highest percentage of the affected area (*p* ≤ 0.05) corresponded to the treatments with the greatest output power (100 W) and longer holding times (120 and 180 s), representing the affected surface around 15% of the testa. As the output power and holding time were lower, the damage statistically decreased, so the treatments with 20 W applied during 60 and 120 s did not present differences (*p* ≤ 0.05) compared to the control. The brown tone of the water (of increasing darkness with the increasing output powers and/or holding times; Figure 2) indicates that it is related to the corresponding degradation of the testa.

### 2.3. Imbibition

The initial seed moisture content was 7.9 ± 0.05%, similar to that of the other lots of seeds used in the other studies carried out by our research team [11,25], and they are within the range usually recommended (5 and 8%, depending on the species) for adequate conservation of the seeds [26,27,28].

**Figure 4:**
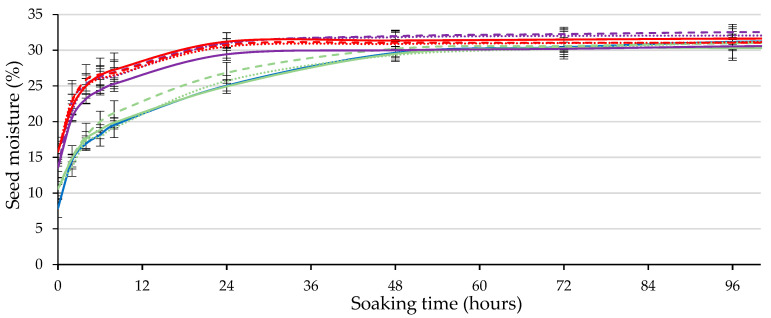
Time course of seed moisture content along the soaking period, considering the output power (control (blue), 20 W (green), 50 W (violet) and 100 W (red)) and the holding time of ultrasound stimulation (60 s (solid), 120 s (dashed) and 180 s min (dotted lines)). Error bars represent the standard error.

The seed moisture content along the soaking period (Figure 4) followed the first two phases of the typical triphasic water uptake model in seed germination [11]. First (phase I of germination; imbibition itself), the water uptake was initially rapid, followed by a slower wetting step. At the end of phase I (from the first to the second day, depending on the seed treatment), the water uptake stopped as the seed entered the lag phase of germination (phase II), in which the metabolism was already supposedly active. 

The seed moisture content increased quickly during the first 24 h of soaking, stabilizing in the seeds stimulated with 50 and 100 W (Figure 4). In contrast, water uptake continued until the second day in control (intact) and in seeds subjected to 20 W. The moisture content of the seeds stimulated with 50 and 100 W was statistically higher (*p* ≤ 0.05; Table 1) than that of the seeds stimulated with 20 W and the control seeds after soaking for 8 and 24 h. When the seed moisture is compared at 48 h of soaking, there were no statistical differences (*p* ≤ 0.05; Table 1) between treatments, reaching a seed moisture content of approximately 31% in all cases. This moisture content is sufficient for the efficient germination of caper seeds [11,25].

These results are in agreement with those reported in the literature. Ranjbari et al. [29] stated that in the initial stages of soaking, the treated seeds absorbed water faster than the control seeds and related this phenomenon to the cavitation process produced by the sonicator. Lo Porto et al. [18] indicated that when soybean seeds were treated with ultrasounds, their water uptake increased without modifying the seed coat’s morphology and wettability, but inducing minor chemical changes of the outer part of the seed coat. Ding et al. [30] indicated that power ultrasound produced micro-openings on the surface of rice grains, which provided new pathways for water to enter, thus enhancing the hydration process. Miano et al. [31] found changes in the micro-structure of the seeds, increasing the seed porosity and improving the mass transfer among the seed tissues.

In general, water uptake in seeds occurs through the seed coat. However, in some seeds, the initial water uptake occurs in specific locations or through inherent structural features in the surrounding tissues, such as the micropylar region in the tobacco seeds, corn and some legumes. In seeds of western white pine (*Pinus monticola* Douglas ex D. Don), the micropyle is impermeable to water, which enters the seed through the surrounding testa [32]. In all cases, during the imbibition process, the seed whole is not moistened simultaneously, and there is a sharp limit of water content between wet cells and those about to get wet [32].

Previous studies determined that water uptake in caper seeds occurs through the hilar region [11]. However, it remains to be determined whether, in seeds treated with ultrasounds, it occurred through the scarified surface of the testa or the hilar region, as in control seeds. The hilar region in the caper seed coat contains two scars, the hilum (scar corresponding to the funiculus of the ovule) and the micropyle (scar corresponding to the micropyle of the ovule).

Figure S2 shows that the moistening of both control and ultrasonicated seeds begins in the hilar region, with the extra hilar region being impermeable. The hilar region is comported as a “water channel”, and according to Foschi et al. [11], caper seeds do not have a strictly waterproof coat and can absorb water without breaking the seed coat. On the other hand, although the ultrasonication has scarified a part of the testa, the tegmen has prevented the uptake of water through it; however, it seems that the tegmen (and the water channel) can become wet, which may be confirmed by the increase in seed water content caused by ultrasonication (Table 1).

While control seeds after 48 h of soaking do not show any coloration, the ultrasonicated seeds with 20 W show the hilar region already colored in blue by the methylene blue solution (Figure S2). On the other hand, the seeds treated with higher output powers show the hilar region colored after 8 h of soaking. Specifically, the seeds treated with higher output powers and holding times show an important part of the embryo colored after 48 h of soaking. This moistening pattern agrees with the previously analyzed moisture contents (Table 1) in the sense that the most potent treatments (in output power and holding time) produced faster imbibition.

### 2.4. Seed Viability

**Figure 5:**
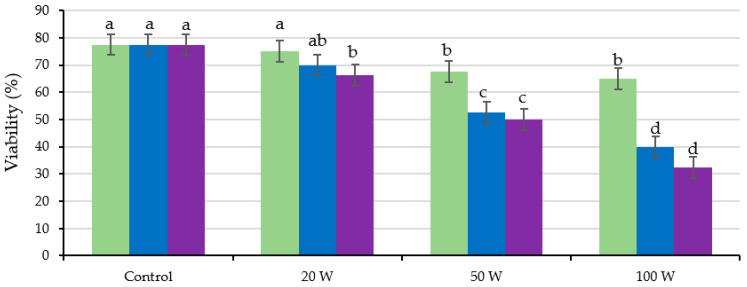
Analysis of the significant interaction of the analysis of variance presented in Table 2 between the output power (Control, 20, 50 and 100 W) and the holding time (60 (green), 120 (blue) and 180 s (violet)) of the ultrasonication effect on the viability (%; sound + weak but viable tissues) of the seeds. Average values of four replicates. Different letters indicate significant differences according to the LSD test (*p* ≤ 0.05), which are represented by the error bars.

The initial viability of the seed lot used in this study was 77.5%, decreasing with ultrasonication as both output power and holding time increased (Table 2; Figure 5) so that the lowest viability (*p* ≤ 0.05) corresponded to the higher power treatments (50 and 100 W) and to the longer holding times (120 and 180 s). Seed viability was also significantly influenced by the interaction of output power and holding time (*p* ≤ 0.05; Table 2; Figure 5). Seed viability decreases with ultrasonication, just slightly (not significantly) with the lowest output power (20 W) applied for the shortest time (60 s), and to a greater extent with increasing both output power and holding time, so that the lowest viability (*p* ≤ 0.05) corresponded to the highest power treatments (50 and 100 W) with the most extended sonication times (120 and 180 s). These results agree with the damage produced in the testa of the seeds (Figure 3) and with the water EC and temperature (Figure 2). Treatments with the highest output power and holding time were the ones that caused the greatest damage to the testa leading, in turn, to the greatest water EC and temperature, which could be related to the loss of seed viability. When analyzing only the sound seeds, both factors significantly affected it (output power and holding time, *p* ≤ 0.01 and *p* ≤ 0.05, respectively), but not their interaction (Table 2). The percentage of sound seeds was statistically reduced compared to the control (*p* ≤ 0.05) even by the lowest power applied. 

### 2.5. Seed Germination

All the analyzed factors, output power and holding time of ultrasonication, the solution used to moisten the substrate in the germination test and their interactions influenced the germination percentage (*p* ≤ 0.01; Table 3 and Table 4). The output power applied and the solution used to moisten the substrate and their interaction explain 21.2%, 49.0% and 23.6%, respectively, of the variability of the test, indicating the importance of both factors. It is observed that germination statistically decreased as the output power and holding time increased, and its value was much higher when the GA_3_ solution was used in the test than when water was applied. Regarding the output power and solution interaction, the significance is related to the low germination obtained with the 100 W treatment, regardless of whether the seeds were moistened with the GA_3_ solution or with water, obtaining similar germination percentages in both cases. The positive results obtained by adding GA_3_ to the substrate coincide with the previous studies by the research team [5,9,11,25], in which the addition of GA_3_ increased germination.

On the other hand, the germination percentage obtained in all the combinations with 100 W of output power (applied during 60, 120 and 180 s and using both water and GA_3_ solution to moisten the substrate in the germination test) was lower than 10%. The germination obtained was also lower than 10% when water was used to wet the substrate in any combination of output powers and holding times. This result agrees with those reported by the research team [33,34,35], who obtained high germination percentages with different scarification methods, but only when they were followed by the addition of a GA_3_ solution. The low germination prevented the determination of the germination curves for these treatments; thus, the germination parameters were only analyzed for the seeds treated with 20 and 50 W and moistened with GA_3_ during the germination test. 

The coefficients of determination (R^2^) obtained for the germination data fitted to the logistic function (*p* ≤ 0.01) were greater than 0.99, for all of the four replicates from the different combinations of variation sources, allowing the use of the variable *A* (final germination percentage; instead of *G*), as well as other variables, such as *Gt*_50_ (number of days needed to reach 50% of the final germination) and *k*/2 (average germination rate; d^−1^), as performed in previous studies of caper seed germination [7,33]. 

**Figure 6:**
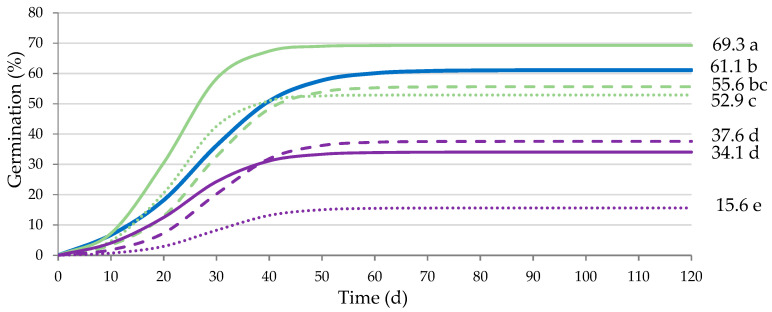
Analysis of the significant interaction of the analysis of variance in Table 5 between the output power (control (blue), 20 W (green) and 50 W (violet)) and the holding time of ultrasounds stimulation (60 s (solid), 120 s (dashed) and 180 s (dotted lines)) on the final germination percentage (*A*). Average values of four replicates. Different letters indicate significant differences according to the LSD test (*p* ≤ 0.05).

Significant differences were obtained for the analyzed factors, output power and holding time and their interaction (*p* ≤ 0.01; Table 5), representing 76.6%, 8.5% and 8.6%, respectively, of the variability of *A* in the germination test. This indicates that output power is the factor causing a greater effect. Given the statistical significance (*p* ≤ 0.01) of the interaction, it is presented in Figure 6.

Seeds ultrasonicated for 60 s at 20 W power obtained a statistically higher *A* (*p* ≤ 0.05) than control seeds, also reducing *Gt*_50_ (from 27.2 to 21.6 d). *A* was significantly affected (*p* ≤ 0.01) by the interaction between the power and the holding time since ultrasonication of 20 W for 60 s was the only treatment that increased *A*, so when the treatment was extended to 120 and 180 s, *A* decreased, being lower than that of the control seeds (*p* ≤ 0.05). *k*/2 was only affected by the output power (*p* ≤ 0.01), resulting in an acceleration of germination when the seeds were treated with ultrasounds compared to the control.

These results coincide with those obtained by Pascual et al. [5], who applying ultrasonication in a bath (for 30 min, using an ultrasonic apparatus P. Selecta Ultrasons, model 513, 150 W, 40 kHz) obtained an increase in germination (up to 71.7%, on average) in caper seeds that presented (on average) a germination percentage of 48%, when they were not treated. Foschi et al. [34] reported that irradiation of caper seeds with a He-Ne laser for short exposure times improved the germination percentage (about 13%) of caper seeds previously soaked in water, with the application of a GA_3_ solution to the germination substrate. This irradiation is a time demanding treatment, as the seeds have to be irradiated one by one, since this He-Ne laser has a circular beam of 0.7 mm in diameter. Juan et al. [35] obtained that exposing caper seeds to magnetic fields increased their imbibition compared to control seeds, and they reported a positive (although not statistically significant) effect of magnetic field exposure on seed germination, which only occurred with the addition of a GA_3_ solution to the substrate. Thus, it can be stated that the herein presented results coincide with those obtained by this team in previous studies, using chemical scarification [33], irradiation with a He-Ne laser [34], and the exposure to magnetic fields [35]. In all these studies, a slight increase in the germination percentage has been obtained when any of these scarification treatments have been carried out together with the addition of GA_3_ compared to those obtained with the sole addition of the GA_3_ solution. However, neither of these treatments replace the action of GA_3_ but rather complemented it.

The germination results also coincide with those reported by López-Ribera and Vicient [14] in the sense that short ultrasonic stimulation (for <1 min) generated with a 45 kHz ultrasonic bath significantly increased the germination of *A*. *thaliana* seeds; however, they did not observe differences in the germination rate when using recently collected seeds that shown a germination percentage close to 90%. On the other hand, longer sonication treatments led to a decline in germination, as in the present study. Babaei-Ghaghelestany et al. [15], in order to break seed dormancy of *Chenopodium album*, applied ultrasonic waves with a frequency of 35 kHz for 5, 10, 15 and 30 min, and they observed that ultrasound waves enhanced the germination percentage, obtaining the greatest germination percentage when seeds were sonicated for 15 min.

The scientific literature relates the effects of ultrasonication on seeds to some mechanisms such as (i) the increase in the seed coat porosity that results in greater absorption of water during hydration, (ii) a slight oscillation of particles due to the movement of waves through tissues, which can lead to a rupture of the cell walls of the seeds, which increases the absorption of water by the seed, and (iii) the intensification of the mass transfer that improves the mobilization of nutrients from the endosperm by breaking the cell membrane [31,36]. Yaldagard et al. [13] stated that the ultrasonication (at 20 kHz on the ultrasonic generator in three different ultrasonic output powers and three holding times) resulted in better and faster germination on barley seeds (*p* ≤ 0.01) due to both the fragmentation of the seed coat and the enlargement of its pore size, improving their hydration, which increased alpha-amylase activity causing their faster germination.

As previously mentioned, the water used for ultrasonication acquired a brown hue, their EC increased, and the testa of the seeds was scarified up to around 15% of their surface (Figure 4). Initially (approximately up to 24 h), ultrasonication stimulated the imbibition process (Figure 5), but there were no differences in the statistical analyzes carried out after 48 h of seed soaking (Table 1). Imbibition in both control and ultrasonicated seeds occurred through the hilar region and not through the tegmen (Figure S2), even though a fraction of the testa had been scarified (Figure 3). Therefore, it seems that ultrasound waves may fragment the hilar region of the seed coat and/or increase its pore size, as indicated by Yaldagard et al. [13] for barley seeds, which led to faster imbibition.

During the seed imbibition, if the hydration occurs slowly, the membranes can reorganize and reach their original structure, increasing the integrity of the membrane. However, if the hydration is too fast, it can cause the leakage of sugars, amino acids and minerals [37]. In the present study, the ultrasound treatment has caused hydration to be faster, to a greater extent, at 50 and 100 W, anticipating the maximum moisture level 24 h compared to the control seeds and those ultrasonicated at 20 W. The water used in the ultrasonication took on a brown hue (Figure 2) of increasing darkness coinciding with the increase in treatment output power and/or holding time, and at the same time, both its EC and its temperature increased (Figure 2). These effects are related to the evident degradation of part of the testa, but they could also be due to the leaching of sugars, amino acids and minerals and to the metabolism caused by a different enzymatic activity, but this should be verified in subsequent studies. It does not seem probable that the acceleration of the hydration process causes the leaching of nutrients, although the high temperatures could affect the enzymatic activity and, therefore, the metabolism of the reserves, which, together with the rupture of the cell walls, could contribute to the decrease of the viability and the percentage of germination of the seeds obtained with the higher output powers and/or holding times (Table 2, Table 3, Table 4 and Table 5).

Wang et al. [38] studied the effects of ultrasonication on germination and seedling growth in switchgrass (*Panicum virgatum*) using an orthogonal matrix design. Among the studied factors (sonication time, temperature and output power), sonication temperature had the largest effect on germination, and temperatures around 40 °C provided the greatest germination percentage, decreasing with increasing temperature. These authors concluded that ultrasonic treatments could have both positive and negative effects on seeds and that, when the output powers, holding times and consequently temperatures used are higher than the corresponding optimal levels, physical or chemical damage may occur due to ultrasonic waves. In the present study, the ultrasound output power of 20 W and the holding time of 60 s provided the greatest germination percentage, reaching 24 °C during the ultrasound application. When the output power and/or holding time were higher, the temperature increased (up to 53.5 °C), and the germination percentage decreased (up to 16%), probably as consequence of the physical or chemical damage produced by the bubble collapse.

**Figure 7:**
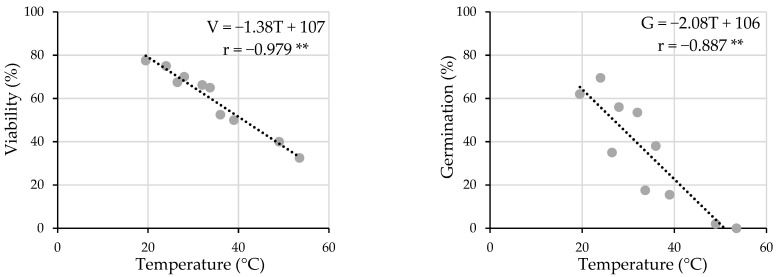
Viability–temperature relationship (**left**) and germination (*G* with GA_3_ application; (**right**))–temperature relationship. **: significance level *p* ≤ 0.01.

Figure 7 shows a high lineal and negative correlation between both viability and germination (*G* when GA_3_ is applied) with the temperature reached during the sonication treatment. In both cases, the correlation is statistically significant (*p* ≤ 0.01), and the corresponding correlation coefficients are −0.98 and −0.89. As seen in the figure, temperatures above 40 °C considerably reduce the seed viability and, in a greater measure, the germination, practically nullifying it.

Analyzing the viability of the seeds that had not germinated after 120 days, it can be stated that those treated with 50 W are all not viable. For control seeds and those subjected to 20 W for 120 and 180 s, the viability of the non-germinated seeds ranged from 2.5% to 5.0%, indicating that slightly higher percentages of seeds could have germinated with more extended germination periods. However, when the seeds were treated with 20 W for 60 s, there were no viable seeds after 120 d, indicating that all the viable seeds germinated. Thus, it can be stated that subjecting the seeds to 20 W for 60 s encourages the germination of all viable seeds.

Foschi et al. [4] stated that adding gibberellins to the substrate in the caper seed germination tests increases the embryonic growth potential so that it can overcome the mechanical resistance imposed by the testa and therefore crack it without weakening it. Probably, according to Yaldagard et al. [13], the fragmentation of the seed coat and/or the enlargement of its pore size in the hilar region caused by ultrasonication led to faster imbibition. However, it could also have weakened the seminal coat in the hilar region and, consequently, improved the germination process, increasing *G*. This is what seems to have happened in the case of ultrasonication with 20 W for 1 min, in which ultrasonication has not replaced the action of GA_3_ but rather has complemented it. Yaldagard et al. [13] also related better seed hydration with increased alpha-amylase activity, which led to a faster germination of barley seeds, but it should be tested for caper seeds in future studies. 

## 3. Materials and Methods

### 3.1. Plant Material

The caper seeds used in the study were collected in September 2020 from a plot in Llíria (Valencia, Spain; 39°38′07.8″ N 0°35′53.4″ W). The viability, ultrasonication, imbibition and germination tests were carried out in April 2021, when the seeds were 6 months old.

The seeds were extracted from ripe fruits collected on the day of their dehiscence and from fruits located immediately before and after it, and they were cleaned with tap water. The mature seeds were separated by the decantation method [39]. To prevent future infections, they were disinfected with a 25% sodium hypochlorite solution (from a concentrated solution of 37 g L^−1^ of active chlorine) for 2 min, followed by three washes with ultrapure water. The seeds were dried and stored in a closed, airtight glass container at 7 °C until the trials were carried out.

### 3.2. Ultrasonication

A Bioblock ultrasonic processor (sonicator), vibra cell model 75115 (Bioblock Scientific, IIIkrich, France), with a CV334 probe, was used. This sonicator works with a frequency of 20 kHz and a maximum output power of 500 W, making it possible to vary the amplitude from 0 to 100%. 

First, before the treatments, the sonicator was calibrated, for which three different amplitudes (25, 50 and 75%) and four holding times (30, 60, 120 and 300 s) were tested. For calibration, 100 mL glass beakers with 80 mL of ultrapure water (hereinafter water) and 50 seeds were used. The calibration was carried out to determine the output power at which each amplitude works and, at the same time, to serve as a support to select the combinations of wave amplitude and holding time to be applied, depending on both temperature and seed viability reached with the different combinations. The energy recorded by the sonicator was used to determine the corresponding power for each holding time. After applying each amplitude–time combination, the water’s temperature and EC were measured using a handheld EC meter (Eutech Cond 6+; Eutech Instruments Pte Ltd.; Waltham, MA, USA), and the seed viability was determined as explained afterwards.

Based on the results of the calibration, it was decided to perform the treatments corresponding to the following combinations: output powers of 20, 50 and 100 W (corresponding to the wave amplitudes of 20%, 50% and 75%) for 60, 120 and 180 s, as well as a control treatment (not subjected to the ultrasounds). After ultrasound treatments were applied, the seed coat’s disruption level was determined, and the imbibition, viability and germination tests were performed.

### 3.3. Disruption of the Seed Coat

As the cavitation can damage the seed coat or create fissures and pores, the percentage of disruption that the seed coat presented after the ultrasound treatments was determined as the percentage of the damaged area in relation to the total seed coat area. These areas were determined from a photographic record taken by a photomicroscope (U500X Digital Microscope; Cooling Tech, Shenzhen, China), and the ImageJ program [40] was used to perform the surface measurements. It was determined in four repetitions of ten seeds each for each treatment.

### 3.4. Imbibition

The imbibition test was performed according to Ma et al. [41] and Juan [7]. After the ultrasound treatments, the seeds were weighed, and their moisture content determined, and then they were soaked in water (10 mL) in glass test tubes at laboratory room conditions. Four replications of ten seeds per treatment were considered. The moisture content of the seeds was determined according to the International Rules for Seed Analysis [42]. Seed weight was determined every 2 h during the first 8 h of imbibition and then every 24 h for 8 days. Seeds were blotted with a paper towel, immediately weighed on a precision balance (Sartorius, model B 120S, Barcelona, Spain) and returned to the test tubes immediately after. Dry weight was determined at each stablished period, by drying the seeds (four samples per treatment) for 48 h at 103 °C in a forced-air oven (Selecta 297; Selecta, Barcelona, Spain). The daily seed moisture content was calculated (Equation (1)) on a fresh mass basis [42]:

(1) $$\mathrm{Seed}\;\mathrm{moisture}=100\;\frac{\left(\mathrm{Fresh}\;\mathrm{weight}\;-\;\mathrm{Dry}\;\mathrm{weight}\right)}{\mathrm{Fresh}\;\mathrm{weight}}$$

Since water is colorless, the advance waterfront within the seeds was determined parallel to the imbibition test, by soaking the seeds in 5 mL of a methylene blue solution for microscopy (Scharlau, Sentmenat, Spain) in test tubes. At each stablished period, seeds were cut in half to observe the entry of the dye inside them, and a photographic record (Figure S2) was taken by a photomicroscope (U500X Digital Microscope; Cooling Tech, Shenzhen, China). Four replications of ten seeds per treatment were considered.

### 3.5. Seed Viability

The tetrazolium topographic test was used to determine the seed viability [43]. For preconditioning, seeds were soaked in water at room temperature for 18 h; then, they were pierced opposite the micropyle and immersed in a 1% tetrazolium solution (Tetrazolium Red. 2,3,5-Triphenyltetrazolium chloride; Sigma, Barcelona, Spain) in the dark, at 30 ± 1 °C, inside a growth chamber (model Zimbueze, Seville, Spain) for another 18 h [43]. After this period, seeds were cut longitudinally to evaluate the staining in the tissues, taking a photographic record with the already mentioned photomicroscope. Four replications of fifty seeds each were performed. A seed was considered viable when the maximum area of unstained tissue was the radicle tip and when it is expected to germinate under normal conditions [44]. Seeds with sound tissues (gradual and uniform spots on exposed surfaces) and weak but viable tissues (stain grayish red or brighter red than normal) are considered viable [44].

### 3.6. Seed Germination

Germination tests were carried out following the Between Paper (BP) method [42], within Petri dishes (9 cm). The samples consisted of 400 seeds (4 replications of 100 seeds). Two solutions were used to moisten the paper: a GA_3_ solution (500 mg L^−1^; Semefil L; Nufarm, Melbourne, Australia) and water. To avoid fungal contamination, 2 g L^−1^ of Captan (Captan 50; Bayer, Leverkusen, Germany) was added to the solutions. Germination took place for a maximum of 120 days in a growth chamber (model Zimbueze, Seville, Spain) which conditions were 30 ± 1/20 ± 1 °C, 85 ± 1% relative humidity, and a photoperiod of 12 h (81.1 μmol m^−2^ s ^−1^).

A seed was considered germinated when the emerged radicle reached 2 mm long. The difference between the maximum and minimum germination percentages of the four replications did never exceed the tolerance level established [42]. For analyzing the germination curves, the model applied to each repetition is that of the logistic function (Equation (2)) [45]:

(2) $$G=\frac{A}{{1+e}^{\left(\beta \;-\;kt\right)}}$$

with *G* being the accumulated germination (%), *A* being the maximum germination (%), t being the germination period (d), *β* being a parameter referring to the position of the curve relative to the time axis and *k* being a velocity parameter. The last two values are used to calculate the number of days needed to reach 50% *A* (*β*/k = *Gt*_50_) and the average relative rate of cumulative germination (*k*/2, d^−1^).

### 3.7. Statistical Analysis

Analysis of variance (ANOVA) was used to analyze the results with Statgraphics Centurion 18 software [46]. The differences were considered significant for a probability of *p* ≤ 0.05%. The means were separated by the Fisher’s minimum significant differences test (LSD test) at *p* ≤ 0.05. The percentage data were arcsin √x transformed before analysis to accomplish the normality assumption. The normality distribution was analyzed by verifying the residuals’ normal distribution [47] by the Shapiro–Wilk test [46]. 

## 4. Conclusions

Ultrasonic treatments can have both positive and negative effects on caper seeds; when the output power and sonication holding times are higher than the corresponding optimal levels, damage may occur due to ultrasonic waves. Ultrasonication produces faster initial imbibition, but after 48 h of soaking in water, seed moisture does not present differences compared to non-sonicated seeds. Ultrasonication leads to scarification of the testa but does not affect the tegmen, so moistening occurs through the hilar region, as in control seeds. A high linear and negative correlation between the germination of the seeds with the temperature reached during the sonication treatment was obtained, so that temperatures above 40 °C practically annulled the germination. In the present study, the ultrasound output power of 20 W for 60 s provided the greatest germination percentage, being the only treatment that statistically improves germination in relation to control seeds. When the output power and/or holding time were higher, the temperature increased, and the germination percentage statistically decreased.

## Figures and Tables

**Table 1:** Effect of the output power (W) and holding time (s) of ultrasonic treatments on seed moisture content during the seed soaking in water.

	0 h	8 h	24 h	96 h
Output power (P)				
Control	7.9 c	19.6 b	25.0 b	31.2
20	10.6 b	20.4 b	25.8 b	30.7
50	13.5 a	26.2 a	30.9 a	31.7
100	15.8 a	26.9 a	30.8 a	31.3
Holding time (T)				
60	11.3	23.0	27.7	30.9
120	12.0	23.1	28.2	31.4
180	12.6	23.6	28.6	31.4
Analysis of variance				
Factors (degrees of freedom)	% Sum of squares			
P (3)	54.6 **	68.6 **	68.6 **	2.6 NS
T (2)	1.6 NS	0.5 NS	1.3 NS	1.4 NS
P × T (6)	1.0 NS	0.9 NS	2.8 NS	3.9 NS
Residual (36)	42.8	30.0	27.2	92.0
Standard deviation (^+^)	3.0	2.5	2.0	2.4

Different letters in the same column within each factor indicate significant differences (*p* ≤ 0.05) according to the LSD test. **: significance level *p* ≤ 0.01, NS: not significant. (^+^) The standard deviation was calculated as the square root of the residual mean square.

**Table 2:** Effect of the output power (W) and the holding time (s) of the ultrasonication on the viability (%) of the seeds, considering both sound tissues and sound + weak but viable tissues.

	Sound	Sound + Weak but Viable
Output power (P)		
Control	55.00 a	77.50 a
20	42.50 b	70.42 a
50	27.50 c	56.67 b
100	24.17 c	45.83 c
Holding time (T)		
60	43.75 a	71.25 a
120	35.63 ab	60.00 b
180	32.50 b	56.56 b
Analysis of variance		
Factors (degrees of freedom)	% Sum of squares	
P (3)	53.93 **	53.29 **
T (2)	7.97 *	13.99 **
P × T (6)	3.97 NS	9.62 *
Residual (36)	34.14	23.10
Standard Deviation (^+^)	11.33	9.31

Different letters in the same column within each factor indicate significant differences (*p* ≤ 0.05) according to the LSD test. **: significance level *p* ≤ 0.01, *: significance level *p* ≤ 0.05, NS: not significant. (^+^) The standard deviation was calculated as the square root of the residual mean square.

**Table 3:** Effect of the output power (W) and the holding time (s) of the ultrasonication, and the use of the GA_3_ solution to moisten the substrate of germination test, on the germination percentage (*G*, %) of the seeds.

	*G* (%)
Output power (P)	
Control	32.8 a
20 W	32.2 a
50 W	19.2 b
100 W	5.4 c
Holding time (T)	
60	26.4 a
120	22.8 b
180	17.9 c
Solution (S)	
Water	5.3 b
GA_3_	39.4 a
Analysis of Variance	
Factors (degrees of freedom)	% Sum of squares
P (3)	21.2 **
T (2)	2.0 **
S (1)	49.0 **
P × T (6)	1.2 **
P × S (3)	23.6 **
T × S (2)	0.7 **
P × T × S (6)	1.0 **
Residual (72)	1.4
Standard deviation (^+^)	3.3

Different letters in the same column within each factor indicate significant differences (*p* ≤ 0.05) according to the LSD test. **: significance level *p* ≤ 0.01. (^+^) The standard deviation was calculated as the square root of the residual mean square.

**Table 4:** Analysis of the significant interactions of the analysis of variance in Table 3: output power (W) and holding time (s); output power and the solution used to moisten the substrate of germination test; holding time and solution on the germination percentage (*G*) of the seeds. Average values of four replicates.

Power	Time	*G*(%)	Power	Solution	*G*(%)	Time	Solution	*G*(%)
Control	-	32.8 b	Control	GA_3_	62.0 a	60	GA_3_	46.0 a
20	60	36.8 a	20	GA_3_	59.7 a	120	GA_3_	39.5 b
20	120	32.3 b	50	GA_3_	29.5 b	180	GA_3_	32.8 c
20	180	27.5 c	100	GA_3_	6.5 cd	60	Water	6.8 d
50	60	23.0 d	Control	Water	3.5 e	120	Water	6.1 d
50	120	23.5 d	20	Water	4.7 de	180	Water	3.1 e
50	180	11.0 e	50	Water	8.8 c	LSD (*p* ≤ 0.05)		2.3
100	60	13.0 e	100	Water	4.3 de			
100	120	2.8 f	LSD (*p* ≤ 0.05)		2.69			
100	180	0.5 f						
LSD (*p* ≤ 0.05)		3.3						

Different letters in the same column indicate significant differences (*p* ≤ 0.05) according to the LSD test.

**Table 5:** Effect of the output power (W) and the holding time (s) of the ultrasonication on the germination variables: final germination (*A*), number of days needed to reach 50% of the final germination (*Gt*_50_) and average germination rate (*k*/2; d^−1^). Average values of four replicates.

	*A* (%)	*Gt*_50_	*k/2*
Output power (P)			
Control	61.1 a	27.2 a	0.06 c
20 W	59.3 a	23.9 b	0.09 a
50 W	29.1 b	27.3 a	0.08 b
Holding time (T)			
60	54.8 a	24.2 b	0.08
120	51.4 a	28.0 a	0.07
180	43.2 b	26.2 ab	0.08
Analysis of variance			
Factors (degrees of freedom)	% sum of squares		
P (2)	76.6 **	18.8 *	41.6 **
T(2)	8.5 **	18.1 *	1.9 NS
P × T (4)	8.6 **	16.4 NS	6.4 NS
Residual (27)	6.3	46.7	50.0
Standard deviation (^+^)	4.9	2.9	0.01

Different letters in the same column within each factor indicate significant differences (*p* ≤ 0.05) according to the LSD test. **: significance level *p* ≤ 0.01, *: significance level *p* ≤ 0.05, NS: not significant. (^+^) The standard deviation was calculated as the square root of the residual mean square.

## Data Availability

Not applicable.

## Supplementary Materials

The following supporting information can be downloaded at: https://www.mdpi.com/article/10.3390/plants12122379/s1, Figure S1: Appearance of 100 ml glass beakers with 80 mL of water and 50 seeds after ultrasonic treatments at powers of 0, 20, 50 and 100 W (from left to right) and a holding time of 60 s; Figure S2: Time course of the staining with the methylene blue solution of the ultrasonicated seeds for 60, 120 and 180 s with the output powers of 20, 50 and 100.
