# Supplementary material for: Effects of High Intensity Ultrasound Stimulation on the Germination Performance of Caper Seeds
Source: Plants (Basel). 2023 Jun 19;12(12):2379. doi: 10.3390/plants12122379 (PMC10302670; doi:10.3390/plants12122379)
Supplement: Supplementary file 1 [file plants-12-02379-s001.zip › plants-2406203-supplementary.pdf]

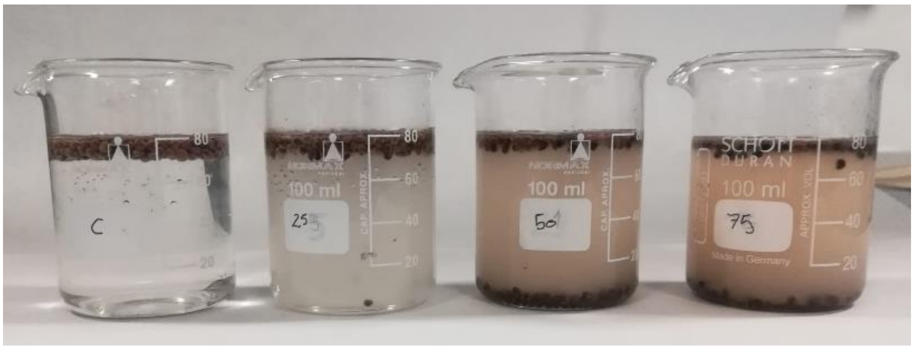

Figure S1. Appearance of 100 mL glass beakers with 80 mL of water and 50 seeds after ultrasonic treatments at powers of 0, 20, 50 and 100 W (from left to right) and a holding time of 60 s.

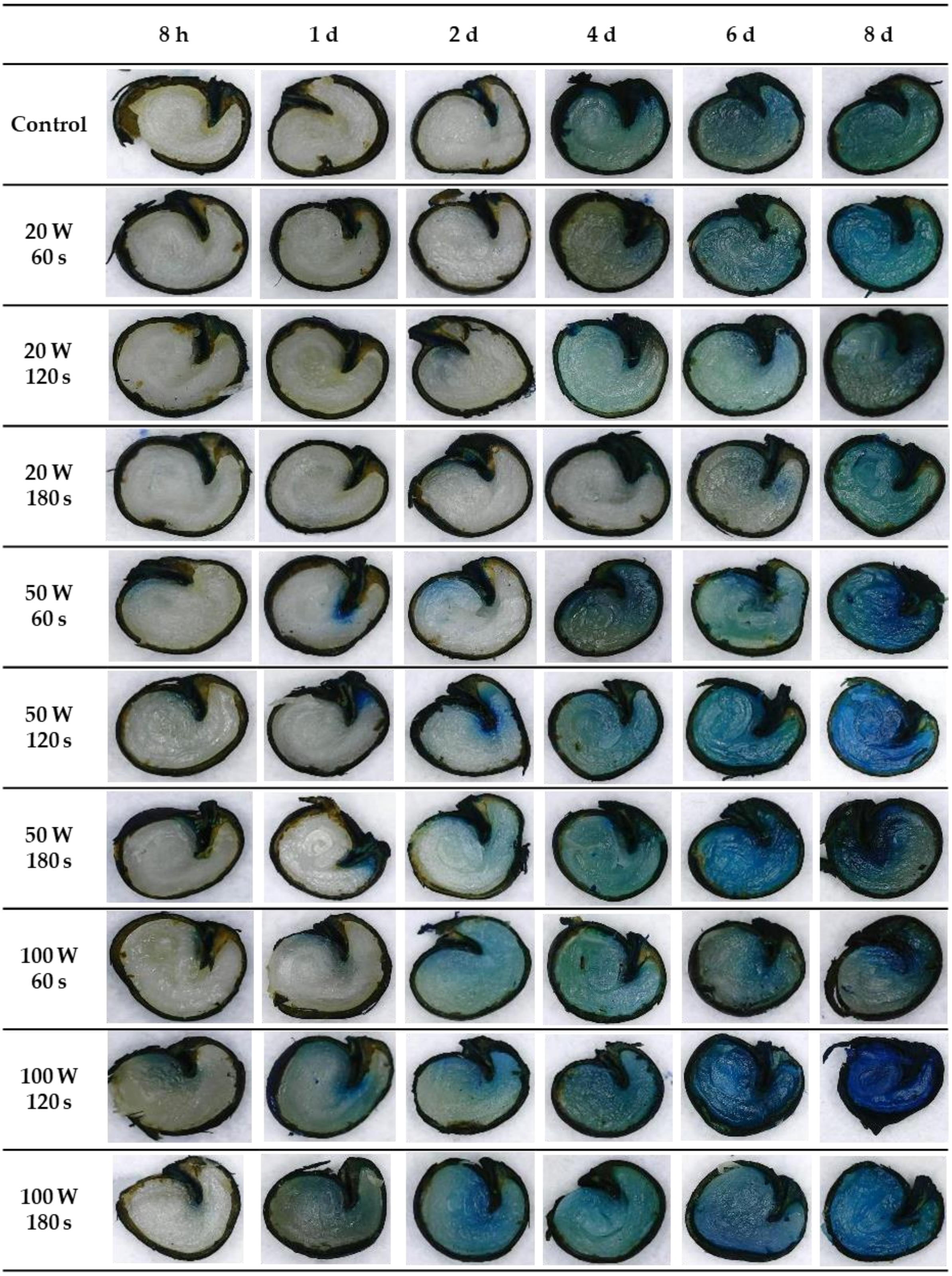

Figure S2. Time course of the staining with the methylene blue solution of the ultrasonicated seeds for 60, 120 and 180 s with the output powers of 20, 50 and 100 W.
